# The road ahead in clinical network neuroscience

**DOI:** 10.1162/netn_a_00103

**Published:** 2019-09-01

**Authors:** Linda Douw, Edwin van Dellen, Alida A. Gouw, Alessandra Griffa, Willem de Haan, Martijn van den Heuvel, Arjan Hillebrand, Piet Van Mieghem, Ida A. Nissen, Willem M. Otte, Yael D. Reijmer, Menno M. Schoonheim, Mario Senden, Elisabeth C. W. van Straaten, Betty M. Tijms, Prejaas Tewarie, Cornelis J. Stam

**Affiliations:** Department of Anatomy and Neuroscience, Amsterdam Neuroscience, Vrije Universiteit Amsterdam, Amsterdam UMC, Amsterdam, The Netherlands; Department of Psychiatry, Brain Center, University Medical Center Utrecht, Utrecht, The Netherlands; Melbourne Neuropsychiatry Centre, University of Melbourne and Melbourne Health, Melbourne, Australia; Department of Neurology, Clinical Neurophysiology and MEG Center, Amsterdam Neuroscience, Vrije Universiteit Amsterdam, Amsterdam UMC, Amsterdam, The Netherlands; Alzheimer Center Amsterdam, Department of Neurology, Amsterdam Neuroscience, Vrije Universiteit Amsterdam, Amsterdam UMC, Amsterdam, The Netherlands; Connectome Lab, Department of Neuroscience, section Complex Trait Genetics, Center for Neurogenomics and Cognitive Research, Amsterdam Neuroscience, Vrije Universiteit Amsterdam, Amsterdam UMC, Amsterdam, The Netherlands; Department of Neurology, Clinical Neurophysiology and MEG Center, Amsterdam Neuroscience, Vrije Universiteit Amsterdam, Amsterdam UMC, Amsterdam, The Netherlands; Alzheimer Center Amsterdam, Department of Neurology, Amsterdam Neuroscience, Vrije Universiteit Amsterdam, Amsterdam UMC, Amsterdam, The Netherlands; Connectome Lab, Department of Neuroscience, section Complex Trait Genetics, Center for Neurogenomics and Cognitive Research, Amsterdam Neuroscience, Vrije Universiteit Amsterdam, Amsterdam UMC, Amsterdam, The Netherlands; Department of Clinical Genetics, Amsterdam Neuroscience, Vrije Universiteit Amsterdam, Amsterdam UMC, Amsterdam, The Netherlands; Department of Neurology, Clinical Neurophysiology and MEG Center, Amsterdam Neuroscience, Vrije Universiteit Amsterdam, Amsterdam UMC, Amsterdam, The Netherlands; Faculty of Electrical Engineering, Mathematics and Computer Science, Delft University of Technology, Delft, The Netherlands; Department of Neurology, Clinical Neurophysiology and MEG Center, Amsterdam Neuroscience, Vrije Universiteit Amsterdam, Amsterdam UMC, Amsterdam, The Netherlands; Biomedical MR Imaging and Spectroscopy Group, Center for Image Sciences, University Medical Center Utrecht and Utrecht University, Utrecht, The Netherlands; Department of Pediatric Neurology, Brain Center, University Medical Center Utrecht, Utrecht, The Netherlands; Department of Neurology, Brain Center, University Medical Center Utrecht, Utrecht, the Netherlands; Department of Anatomy and Neuroscience, Amsterdam Neuroscience, Vrije Universiteit Amsterdam, Amsterdam UMC, Amsterdam, The Netherlands; Department of Cognitive Neuroscience, Faculty of Psychology and Neuroscience, Maastricht University, Maastricht, The Netherlands; Maastricht Brain Imaging Centre, Faculty of Psychology and Neuroscience, Maastricht University, Maastricht, The Netherlands; Department of Neurology, Clinical Neurophysiology and MEG Center, Amsterdam Neuroscience, Vrije Universiteit Amsterdam, Amsterdam UMC, Amsterdam, The Netherlands; Alzheimer Center Amsterdam, Department of Neurology, Amsterdam Neuroscience, Vrije Universiteit Amsterdam, Amsterdam UMC, Amsterdam, The Netherlands; Department of Neurology, Clinical Neurophysiology and MEG Center, Amsterdam Neuroscience, Vrije Universiteit Amsterdam, Amsterdam UMC, Amsterdam, The Netherlands; Department of Neurology, Clinical Neurophysiology and MEG Center, Amsterdam Neuroscience, Vrije Universiteit Amsterdam, Amsterdam UMC, Amsterdam, The Netherlands

**Keywords:** Connectome, Graph analysis, Neuroimaging, Neurophysiology, Computational modeling, Network neuroscience, Clinical application

## Abstract

Clinical network neuroscience, the study of brain network topology in neurological and psychiatric diseases, has become a mainstay field within clinical neuroscience. Being a multidisciplinary group of clinical network neuroscience experts based in The Netherlands, we often discuss the current state of the art and possible avenues for future investigations. These discussions revolve around questions like “How do dynamic processes alter the underlying structural network?” and “Can we use network neuroscience for disease classification?” This opinion paper is an incomplete overview of these discussions and expands on ten questions that may potentially advance the field. By no means intended as a review of the current state of the field, it is instead meant as a conversation starter and source of inspiration to others.

## INTRODUCTION

What are the large-scale network principles governing neuronal communication, cognition, and ultimately human behavior? In history, there have been two main views on the neural correlates of behavior. The first stipulates that the brain consists of separate parts or components, each responsible for a particular function. Proponents of this localizationist view were for instance Franz Joseph Gall and Paul Broca. The opposing view has centered on the unitary, integrative nature of the brain, assuming it impossible to attribute particular functions to either structure or function of particular brain regions. This view was supported by for instance Jean Pierre Flourens and Karl Lashley. The very existence of the journal in which this manuscript is published indicates a new era in neuroscience: After centuries of exploration of these opposing views separately, network neuroscience offers a mathematical framework and model of the brain that combines global integration and local specialization in both structural and functional networks. This framework thereby may be exclusively able to combine the best of both worlds in a quantitative and theory-governed manner.

**Figure 1.**
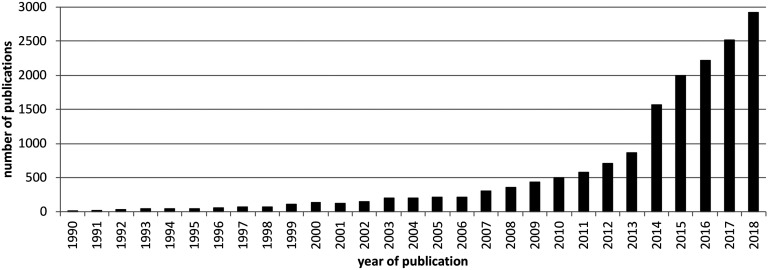
Number of publications on network neuroscience per year between 1990 and 2018

The seminal work of Watts and Strogatz on the structural neuronal organization of the nematode *Caenorhabditis elegans* together with the introduction of power-law and scale-free graphs by Barabasi and Albert (Barabasi & Albert, 1999; Watts & Strogatz, 1998) have founded this new research field, and the number of published works in the field of network neuroscience has been on a steep growth curve ever since (see Figure 1). Recent volumes and reviews have drawn up the current state of the art from multiple perspectives: There are ample overviews of what is currently known about network neuroscience in the context of methodology and network organization (Fornito, Zalesky, & Bullmore, 2016; Sporns, 2010), computational modeling (Bassett, Zurn, & Gold, 2018), and clinical studies (Crossley et al., 2014; Fornito, Bullmore, & Zalesky, 2017; O’Neill et al., 2018; Stam, 2014). However, there is still a need to tackle new challenges and develop novel approaches for future clinical brain network studies.

In this paper, we aim to identify key questions in clinical network neuroscience in the style initiated by Hilbert (the famous mathematical agenda; Hilbert, 1902) and more recently used by Stephan, Bach, et al. (2016) and Stephan, Binder, et al. (2016). It is the product of discussions of the informal group of Netherlands Network Neuroscientists (N^3^), a multidisciplinary collection of scientists who have been working on network neuroscience since its inception. This paper and the questions we address here are by no means meant to be complete or representative and are not intended as a review of the field. Rather, the work is a collection of views on the current state of the field and the most promising and interesting research questions for the near future (see Table 1 for an overview of questions addressed). It naturally falls into two parts: a theoretical section on structural and (dynamic) functional network topology, and a section on future applications of network neuroscience to clinical practice. Our hope is that this paper will spark ideas and facilitate discussions and may be used for inspiration by network neuroscientists, clinicians, and other interested readers.

**Table 1.** Overview of key questions in this piece

**Questions**
*Part I: Theoretical, methodological, and conceptual challenges*
How can we overcome methodological hurdles towards reliable and reproducible applications of clinical network neuroscience?
How do dynamic processes alter the underlying structural network?
What is the role of time-varying dynamics in structure-function coupling?
How can combined computational modeling and experimental work be used in clinical applications?
What are the implications of directionality in the macroscopic brain network?

*Part II: Clinical challenges*
How can we increase our understanding of disease through network trajectories?
What is needed to use brain network characteristics as biomarkers?
Can we use network neuroscience for disease classification?
Can we systematically bridge the gap between brain network interventions in silico and in vivo?
Are evolution and dissolution driving factors in disease connectomics?

## PART I: THEORETICAL, METHODOLOGICAL, AND CONCEPTUAL CHALLENGES

### How can we overcome methodological hurdles towards reliable and reproducible applications of clinical network neuroscience?

An initial major hurdle for clinical network neuroscience is reliability of measures used (Colclough et al., 2016; Jin, Seol, Kim, & Chung, 2011; Noble et al., 2017; Sinke et al., 2018) and reproducibility within subjects (Deuker et al., 2009; Garcés, Martín-Buro, & Maestú, 2016; Hallquist & Hillary, 2019; Telesford et al., 2010). Current methodological challenges in neuroimaging and neurophysiology studies are found on multiple levels, including general neuroimaging-related methodological and statistical issues (Eklund, Nichols, & Knutsson, 2016; Poldrack et al., 2017), as well as (homogenization of) preprocessing pipelines tailored to accurate estimation of both structural and functional connections (Esteban et al., 2019; Kale, Zalesky, Gollo, & Sporns, 2018; Maier-Hein et al., 2017). All these methodological choices currently lack a gold standard, and are rightfully tailored to individual research questions, relating to which some pitfalls are more important than others. Overall, however, the multitude of processing choices diminishes comparability between studies, as different studies almost never use the exact same methodology at all levels, ranging from scanning or recording to structural or functional network reconstruction. As a result, network neuroscience studies are generally weakly reproducible and have limited reliability, while rapid methodological innovations in the field may question the validity of studies performed only a few years ago. Network neuroscientists need to continuously inform themselves about the current state of the art and incorporate advances in overarching methodologies.

Apart from these general methodological challenges, network neuroscience has several methodological challenges that are specific to the field. Generally, longer recording times for functional connectivity studies based on resting-state functional MRI (rsfMRI), electroencephalography (EEG), and magnetoencephalography (MEG) may increase reliability and reproducibility of findings (Birn et al., 2013; Fraschini et al., 2016; Liuzzi et al., 2017), but are possibly limitedly available in practice, particularly in patient populations. Additionally, network neuroscience generally makes use of predefined node definitions that have been critically evaluated in recent years, for example concerning the validity of standard brain atlases (Power, Schlaggar, Lessov-Schlaggar, & Petersen, 2013; Zalesky, Fornito, & Bullmore, 2010). Edge definitions also have inherent issues, such as the optimal measure to quantify structural and functional connectivity per modality (Bonilha et al., 2015; Colclough et al., 2016) and the issue of thresholding approaches, which impacts the estimated topology of the reconstructed functional (van Wijk, Stam, & Daffertshofer, 2010) or structural network (Maier-Hein et al., 2017). Thresholding techniques may also induce more noise to the network, further complicating their use in clinical practice (Zalesky et al., 2016).

Whereas much effort and literature are currently directed at developing better data-driven, computational methods to improve analysis pipelines, theory-inspired approaches may also aid in increasing reliability and reproducibility of network neuroscience. With respect to thresholding for instance, more data-driven approaches include efficiency cost optimization, proportional thresholding, and probabilistic thresholding (De Vico Fallani, Latora, & Chaves, 2017; van den Heuvel et al., 2017; Vasa, Bullmore, & Patel, 2018). A more network-based theoretical strategy towards thresholding is the minimum spanning tree. The idea of this approach is that analysis of a core subgraph or “backbone” may overcome some of the issues of reproducibility and within-subject variability, particularly in heterogeneous populations (Stam et al., 2014; van Dellen et al., 2018), while recapitulating the most important aspects of the underlying system (Tewarie et al., 2015). Keeping these analysis- and preprocessing-related caveats in mind, we now proceed with more conceptual perspectives in the remainder of this review.

### How do dynamic processes alter the underlying structural network?

The brain is a highly plastic organ, which constantly adapts to varying input from the environment and from its own internal perturbations. Many, often complex, molecular mechanisms are involved in homeostatic synaptic plasticity (Pozo & Goda, 2010). In addition, structural plasticity plays a crucial role in maintaining the overall organization of efficient brain networks (Butz & van Ooyen, 2013; Fauth & Tetzlaff, 2016; Yin & Yuan, 2015). Up to now (dynamic) functional connections are usually seen as an effect of the underlying structural connectivity. However, functional communication may also impact the structural connections between any set of brain regions. Studies using techniques such as transcranial direct current stimulation (tDCS) or transcranial magnetic stimulation (TMS), which intercede on the functional communication between neurons noninvasively, have reported on alterations in structural connectivity following functional stimulation (Lindenberg, Nachtigall, Meinzer, Sieg, & Flöel, 2013; Zheng & Schlaug, 2015). This type of functional modulatory influence is also likely to play an important role in the plasticity of brain networks during development, learning, and response to disease. In addition, understanding and predicting the effect of various treatment interventions on multimodal brain networks could benefit from a better understanding of the brain as an “adaptive network,” where the structural network changes as a result of activity or other dynamic processes (de Haan, van Straaten, Gouw, & Stam, 2017).

Network science considers a network as consisting of two essential ingredients: (a) a structure (or graph, topology) that can be associated with “hardware” and (b) a function (or process, related to “software”). This duality between structure and function distinguishes network science from graph theory, which mainly studies the organization of a fixed graph, and systems theory that focuses on dynamic processes. A better understanding of plasticity of brain networks, having both structural and functional components, can be obtained by developing appropriate computational models of adaptive networks, where the function interacts with the structure and vice versa. While adaptive network models are challenging to define because of the interaction of two types of dynamics (i.e., of the nodes and the links) on different timescales, a number of studies have shown the feasibility of this approach. A particularly popular class of dynamic processes on networks, called the “local rule–global emergent behavior” (LrGe) class, describes the collective action of the local rules executed at each node that give rise to complex, emergent global behavior (Van Mieghem & van de Bovenkamp, 2015). Some examples of the LrGe class are epidemic models such as susceptible-infected-susceptible (SIS) and susceptible-infected-recovered (SIR) models and general reaction-diffusion processes (Pastor-Satorras, Castellano, Van Mieghem, & Vespignani, 2015). Adaptive LrGe models may also be a powerful class of model for use in network neuroscience.

So far, more biologically informed models have been used to infer global dynamics from local properties. Indeed, a model of coupled neural masses using a combination of functional (synaptic) and structural plasticity could explain the emergence of modularity and time-dependent recovery from lesions (Stam, Hillebrand, Wang, & Van Mieghem, 2010). A model of homeostatic structural plasticity was introduced by Butz and colleagues, and was shown to be effective in explaining recovery from brain lesions (Butz, van Ooyen, & Worgotter, 2009). It has also been shown that including some form of synaptic plasticity may improve the fit of models of fMRI BOLD signals or oscillatory MEG signals to empirical data (Hellyer, Jachs, Clopath, & Leech, 2016; Rocha, Koçillari, Suweis, Corbetta, & Maritan, 2018). Papadopoulos and colleagues showed that plasticity in a Kuramoto model of coupled oscillators resulted in complex topology of the structural and functional networks (Papadopoulos, Kim, Kurths, & Bassett, 2017). In a computational model of Alzheimer’s disease, inclusion of activity-dependent loss of synaptic strength could explain various features of the evolution of the disease process and the possible functional response to different types of treatment (de Haan, Mott, van Straaten, Scheltens, & Stam, 2012; de Haan et al., 2017).

Typically, computational models of plasticity in brain networks involve systems of coupled harmonic oscillators or neural mass models. Such systems require nonlinear stochastic differential equations and are difficult to analyze mathematically. However, dynamic processes that change the underlying graph have also been studied with more simplistic models. The simplest and most tractable model of the above-described LrGe class is the SIS epidemic spread on a graph, in which nodes can be in two states, namely infected or healthy. The local rule is twofold: If a node is infected, (a) it can infect its direct healthy neighbors with infection rate *β*, and (b) it can heal with rate *δ*. Both the infection and the healing or recovery process are independent. These simple rules lead to complex emergent, global network dynamics characterized by two regimes after a sufficiently long time: either an endemic regime develops, in which the epidemic is still active throughout the network, or a healthy regime exists, where the epidemic has disappeared from the network. The two regimes are separated by a sharp phase transition at a precise ratio *β*/*δ* close to the inverse of the spectral radius of the adjacency matrix of the graph. Nearly all LrGe models feature such high-level description.

As such, an epidemic process on a graph with activity-dependent rewiring that changes the structural network can give rise to very rich behavior, while still allowing to obtain analytical understanding of the model (Gross, Dommar D’lima, & Blasius, 2006). One limitation of this rewiring SIS model used by Gross and colleagues was the fact that the number of links was kept constant. More recently, Guo and colleagues introduced the adaptive susceptible-infected-susceptible (ASIS) model on graphs (Guo, Trajanovski, van de Bovenkamp, Wang, & Van Mieghem, 2013). The ASIS model does not have the limitation of a fixed density, produces rich behavior such as a phase transition and modularity, yet still allows some analytical computation. As a next step, a modification of the ASIS model was introduced to capture the phenomenon of (structural) Hebbian learning (Trajanovski, Guo, & Van Mieghem, 2015). A major challenge for future studies of adaptive brain networks is its integration with more biologically and neurophysiologically realistic models. Ideally, such models of adaptive brain networks could be fitted to the data of individual patients, and serve as the starting point for diagnosis, prediction of treatment effects, and prognosis (Bansal, Nakuci, & Muldoon, 2018).

### What is the role of time-varying dynamics in structure-function coupling?

Structural brain network cores—sets of brain regions and connections occupying a central topological position in the network—are believed to play a key role in facilitating communication and integration of information in the network, thus shaping functional connectivity patterns and underpinning cognitive abilities (Avena-Koenigsberger, Misic, & Sporns, 2017; van den Heuvel & Sporns, 2013; Zamora-López, Chen, Deco, Kringelbach, & Zhou, 2016). The topology of the structural network and the presence of cores constrain the landscape of functional dynamics taking place on the network (Boccaletti, Latora, Moreno, Chavez, & Hwang, 2006). Other properties of the structural network that shape the functional network include the Euclidean distance between regions (Alexander-Bloch et al., 2013), the structural degrees of functionally connected regions (Stam et al., 2016; Tewarie et al., 2014), and detours along the shortest paths in the structural network (Goñi et al., 2014). Computational models that simulate neuronal interactions on anatomical networks can reproduce the emergence of functional networks (Deco, Jirsa, & McIntosh, 2011) and dynamic characteristics of neuroimaging recordings (Hansen, Battaglia, Spiegler, Deco, & Jirsa, 2015).

Although several studies have aimed to formalize a mapping between structural and functional networks, the relationship between the structural and functional dimensions in the brain is only partially understood. While there is no one-to-one correspondence between structural and functional connectivity in the brain, static functional networks can be understood in terms of a weighted sum of direct and indirect paths on the underlying structural networks (Bettinardi et al., 2017; Mehta-Pandejee, Robinson, Henderson, Aquino, & Sarkar, 2017; Meier et al., 2016; Robinson, Henderson, Matar, Riley, & Gray, 2009). However, the temporal, time-varying dimension of functional connectivity is a key aspect of communication models and a basic expression of mental processes (Avena-Koenigsberger et al., 2017; Chang & Glover, 2010; Hutchison et al., 2013; O’Neill et al., 2018). This dimension is ignored in most studies that investigate the structure-function relationship in the brain, mainly because of difficulties in measuring and quantifying dynamics (Griffa et al., 2017; Lurie et al., 2018; Vidaurre et al., 2018). We argue that an empirical understanding of the relation between structural network topology and dynamics of functional connectivity is a fundamental prerequisite for the development (or refinement) of models of brain functioning.

Recent work has begun to explore how time-evolving functional networks during the resting state emerge from a single structural network (Amico et al., 2017; Cabral, Kringelbach, & Deco, 2017; Tewarie et al., 2018). Moreover, task states have been shown to affect directed or effective connectivity, as well as local dynamics exhibited by cortical hub regions (Senden, Reuter, van den Heuvel, Goebel, & Deco, 2017; Senden et al., 2018). For instance, a transition from unstructured to oscillatory behavior exhibited by cortical rich-club regions (i.e., by regions that occupy a central position in the structural network) demarcates task states from rest (Senden et al., 2017). The observed timescale closely matches those that characterize the transition between structured and unstructured functional connectivity configurations as measured in other studies (Glomb, Ponce-Alvarez, Gilson, Ritter, & Deco, 2018), as well as the evolution of performance on diverse tasks (Gilden, Thornton, & Mallon, 1995). This suggests a complex interplay between structural network organization, local dynamics of both core and periphery, functional network formation, and task performance.

Dynamics may vary over multiple timescales. For example, disease and task states occur in the context of a dynamically (and periodically) varying vigilance state driven by the circadian rhythm. Only a handful of studies have investigated the effect of the circadian rhythm on functional coupling during rest in healthy populations (Blautzik et al., 2013; Hodkinson et al., 2014; Koenis et al., 2013). Research on how the circadian rhythm affects the dynamics of brain communication in clinical populations or during task performance appears to be entirely lacking. Given that the circadian rhythm affects attention, learning, and decision-making (cf. Schmidt, Collette, Cajochen, & Peigneux, 2007), the circadian rhythm likely constitutes an important factor that affects brain dynamics and functional network formation in a manner that is also dependent on the underlying structural network. Unraveling the structure-function interplay calls for an integrative approach involving multimodal neuroimaging and computational modeling. A tangible step forward would be to report both structure and function in the setting of within-scan and circadian variation.

### How can combined computational modeling and experimental work be used in clinical applications?

Application of network analysis to human brain data may be useful, but often remains phenomenological in its assessment of brain functioning. Computational modeling helps in formalizing the assumed relationships between structure and function that we seem to observe. In order to identify the mechanisms that underlie the organization of (structural and functional) brain networks and that ultimately lead to behavior, a theoretical framework is needed, based on computational neuroscience (Bassett et al., 2018). However, unlike in physics, the feedback loop between theory and experiment is almost nonexistent in computational network neuroscience. To facilitate modeling studies in network neuroscience, the three steps of denotation, demonstration, and interpretation of models in science are viewed as essential by Hughes (1997). These three steps involve establishing a model that captures relevant aspects of the target system (denotation), studying the model analytically or numerically to demonstrate effects within the model (demonstration), and relating these effects back to the target system as well as validating the model by comparing (simulated) model behavior with empirical observations of the target system (interpretation).

A multitude of simulation models are available that are based on different assumptions, parameters (and corresponding parameter spaces), and dynamics, making the first step of denotation already difficult (Prinz, Bucher, & Marder, 2004). Different models can uphold different oscillatory and network behavior (Abeysuriya et al., 2018; Ashwin, Coombes, & Nicks, 2016; Daffertshofer, Ton, Pietras, Kringelbach, & Deco, 2018): some models may support partial synchronization, whereas others are able to support scale-free synchronization (Daffertshofer et al., 2018). It is thus important to be aware of the question one aims to address (i.e., which aspects of the target system one is interested in) and to choose the model appropriately. Different groups have, for instance, gained important insights from nonbiological or conceptual models (Mišić et al., 2014; Stam et al., 2016), with the argument that the finer details of a model are irrelevant as long as the model is able to simulate realistic behavior on a systems level. For example, dynamic models that belong to the same type or class of models behave similarly near a phase transition (e.g., synchronization and epidemics act similarly around the phase transition). However, these nonbiological models usually do not support multi- or metastability, which is likely the dynamic regime in which the brain operates (Cabral et al., 2014; Kringelbach, McIntosh, Ritter, Jirsa, & Deco, 2015). Temporal fluctuations in functional networks are characterized by formation and dissolution of several subnetworks. Switching between subnetworks can be achieved in systems that support multi- or metastability, and therefore these dynamic regimes could be responsible for the emergence of time-varying fluctuations in empirical connectivity (Cabral et al., 2014; Kringelbach et al., 2015; Tewarie et al., 2018). Simplistic models such as epidemic spreading models/diffusion models usually do not support metastability or multistability and are therefore limited in their ability to explain transitions between network states observed in empirical time-varying data. Even when a computational model fits empirical data, the results and potential insight gained from the model will largely depend on the richness of its supporting dynamics (e.g., limit cycle, chaotic behavior, fixed points), and thus conclusions can be highly biased by a limited repertoire of dynamic regimes. Common behavior and generalizations across models have to some extent been studied in the context of healthy brain networks (Messé, Rudrauf, Benali, & Marrelec, 2014), and may also guide correct interpretation.

The second step, demonstration, is illustrated by Hughes through the following example: “The wave theory of light represents light as a wave motion. It invites us to think about optical phenomena in terms of the propagation of waves, and so to anticipate and explain the behavior of light” (Hughes, 1997, p. S331). In this example, a mathematical model of light as wave propagation could be physically demonstrated as wave motion in a water tank. A clear feedback loop between modeled experiments and empirical data would arguably yield a lot of improvement in the relevance of modeling studies for network neuroscience. Clinical network science offers an opportunity, because the impact of brain disease can be studied over time and compared with model-based prediction. Proven examples are models that predict altered functional dynamics (e.g., Deco et al., 2011; Honey & Sporns, 2008) as a result of structural brain damage, where the modeled dynamics resemble empirical findings (Butz & van Ooyen, 2013; de Haan et al., 2012; Rubinov, McIntosh, Valenzuela, & Breakspear, 2009; van Dellen et al., 2013). The comparison to empirical data can then be used to further refine the underlying model. This is a slow iterative process, which will eventually lead to more insight into mechanisms of neuronal oscillations and connectivity, and disease-related changes thereof.

The last step, interpretation, is to translate these inferences to the target system. In order to be truly synergetic, the observations in our models or algorithms should lead to predictions on the versatility, power, and energy consumption of the human brain, and on the fundamentals that shape cognition and behavior.

### What are the implications of directionality in the macroscopic brain network?

The study of structural and functional brain networks has mainly been based on link existence, leading to undirected networks. Undirected networks can be represented by symmetric matrices (such as the adjacency and Laplacian matrix; see Van Mieghem, 2011). In many cases, the process on the network steers items (e.g., information, packets, traffic, flows) over links in a certain direction and thus defines a direction of link (Van Mieghem, 2018). Often, directed links are represented by an asymmetric adjacency matrix. Unidirectionality is a useful simplification to allow progress in the field: analysis of directed graphs is considerably more complicated than analysis of undirected graphs.

The nodes or vertices of the structural brain network correspond to cortical and/or subcortical regions, and the links or edges between them represent bidirectional physical connections (axonal projections) between regions. A functional connection exists if there is a statistical dependency between regional activity patterns, which is thought to relate to the amount (or probability) of communication taking place between brain areas. Although this bidirectionality probably holds true for certain brain areas that are structurally connected, other functional connections are likely unidirectional (Harriger, van den Heuvel, & Sporns, 2012; Honey, Kotter, Breakspear, & Sporns, 2007; Kale et al., 2018; Scannell, Burns, Hilgetag, O’Neil, & Young, 1999; White, Southgate, Thomson, & Brenner, 1986). On a microscopic level, the structural connections between neurons are considered largely unidirectional, as are functional connections, since action potentials from sending neurons set up postsynaptic potentials in the receiving neurons. Although this in itself does not mean that the interactions between neuronal circuits, that is, macroscopic brain regions, are also necessarily unidirectional, there is experimental evidence that such interactions exist at the macroscopic level as well. Indeed, different brain regions may serve as preferred senders or receivers of information, giving rise to (long-range) feed-forward and feed-back interactions, as well as global patterns of directed functional connectivity (Bastos et al., 2015; Hillebrand et al., 2016). The directionality of functional information transfer may depend on structural network characteristics such as node degree (Moon, Lee, Blain-Moraes, & Mashour, 2015), suggesting that processes such as brain development may dynamically alter directionality patterns.

However, it has proven difficult to develop methods to characterize directionality in the human brain. Structural directionality at the axonal level cannot be accurately studied in humans in vivo. Functionally, different computational approaches estimate directionality, such as dynamic causal modeling, phase transfer entropy, and Granger causality. Not only have the assumptions of these methods themselves been challenged (Seth, Barrett, & Barnett, 2015), it has also been questioned whether they are applicable in certain modalities. In particular, fMRI may not be suitable to estimate directionality because of the low temporal resolution of the signals measured (Smith, Pillai, Chen, & Horwitz, 2012; Wen, Rangarajan, & Ding, 2013). Gilson and colleagues show theoretically and numerically that the use of covariances with both zero and nonzero time shifts is the key to infer directed connectivity in fMRI data (Gilson, Moreno-Bote, Ponce-Alvarez, Ritter, & Deco, 2016). Furthermore, accurate estimation of the underlying directed functional network connectivity requires that the time shift for covariances matches the time constant of the dynamical system, which currently is beyond what we can measure in fMRI.

The nonproven assumption of these methods is that the timing of phases or extent of statistical prediction embedded in the measured functional signals reflects direction in the underlying neuronal interactions. Moreover, the recorded time series are often noisy, the number of data points is limited, and differences in, for example, the signal-to-noise ratios of two time series may lead to erroneous conclusions about directional coupling (Bastos & Schoffelen, 2015). It is not yet clear at what timescales directed interactions occur at the macroscopic level. Estimating brief directed interactions is beyond the capabilities of current macroscopic approaches because of the limited number of data points, yet using longer time-windows might mask the true underlying directionality. Similarly, averaging over trials to boost sensitivity might mask intertrial variability in the preferred direction of interactions. Furthermore, while bivariate coupling estimates may be affected by (hidden) common sources, multivariate measures are dependent on the choice of model order, or require a large number of data points (Bastos & Schoffelen, 2015). Testing against appropriate surrogate data may help to avoid false positives (Paluš & Vejmelka, 2007), but many measures may not be sensitive to true functional interactions because the data cannot be sampled sufficiently within the required time frame.

We therefore need to develop more sensitive and reliable estimators of functional directionality. Until such measures have been developed, should we refrain from including directionality in our estimates of network topology? Directionality may be much more than an additional source of insight into the brain network: viewing macroscopic networks as bidirectional may even lead to erroneous reconstructions of their topology (Kale et al., 2018). The work by Kale and colleagues also suggests that as long as directionality is strictly controlled in terms of false positives, structural directionality should not be ignored, even if the directionality estimator is not perfect: The specificity of directional connections has a larger effect on estimated network topology than their sensitivity. However, it remains an open question whether these conclusions also hold for functional brain networks.

Additionally, perturbed functional directionality patterns have been described in neurological and psychiatric disorders such as Alzheimer’s disease (AD), Parkinson’s disease (PD), and delirium (Boon, Hillebrand, Olde Dubbelink, Stam, & Berendse, 2017; Engels et al., 2017; Numan et al., 2017; van Dellen et al., 2014; van Wijk, Cagnan, Litvak, Kühn, & Friston, 2018), and relate to seizure spread in patients with epilepsy (Wilke, van Drongelen, Kohrman, & He, 2010). The topology of the directed functional network may relate to nodal (i.e., brain regional) vulnerability to pathology: Recent fundamental work suggests that topologically different parts of directed networks have distinct sensitivities to the removal (and addition) of regions and connections (Goltsev, Timár, & Mendes, 2017). Moreover, a stereotypical spreading of pathology in progressive disorders has been observed (e.g., Braak et al., 2003). Misfolded disease-related proteins may transfer transsynaptically from neuron to neuron (Brettschneider, Del Tredici, Lee, & Trojanowski, 2015; Wang et al., 2017), suggesting that disease progression might be predicted on the basis of the topology of the directed functional network. The implications of directional brain networks will therefore likely factor into our understanding of neurological and psychiatric disease.

## PART II: CLINICAL CHALLENGES

### How can we increase our understanding of neurological and psychiatric disease through network trajectories?

The application of network theory to neuroimaging and neurophysiology has uncovered associations between cognitive (dys)functioning and brain network behavior. However, these are mainly cross-sectional findings: Most network neuroscientific studies either demonstrate group differences in the setting of neurological or psychiatric disease, or report on associations between network topology and cognitive functioning at the group level. Indeed, interindividual differences in network organization are much larger than intraindividual variations (Gratton et al., 2018). However, although functional brain network characteristics can be used for personal identification (Demuru et al., 2017; Finn et al., 2015) and are related to normal cognitive functioning, these characteristics may be unrelated to the cognitive deterioration seen in neurodegenerative disease. Cognition and both structural and functional brain network topology may dynamically change concurrently or separately. The very essence of neurological and psychiatric disease from a network neuroscience viewpoint, namely that they induce changing network topology that is in itself dynamic and often progressive, might therefore be obscured in strictly controlled cross-sectional case-control studies. Viewing brain network topology, be it structural or functional, at a single time point as the sole signature of any specific disease may then yield incorrect and ambiguous results (Tijms et al., 2013). Adaptive network models, described in Section I, may incorporate the time-varying interplay between structural and functional connectivity and networks in brain disease. Experimentally, the collection of large cohorts of cross-sectional data (e.g., the Human Connectome Project) should be complemented by efforts and funding to collect high-quality longitudinal datasets of particular patient populations. A longitudinal approach not only is crucial for insight into disease trajectories, but also opens the gates towards network neuroscience–based prognostic biomarkers.

The essence here is that simply including more subjects is not always better than facilitating dense longitudinal measurements in fewer participants, particularly when studying elusive symptoms like cognition and behavior. In healthy subjects, several (small and predominantly functional) longitudinal datasets with high temporal sampling are available (e.g., the MyConnectome project, Poldrack et al., 2015, and the Midnight Scan Club, Gordon et al., 2017) and have yielded important insights into the individuality of brain network signatures (Braga & Buckner, 2017). However, this type of focused longitudinal research has rarely been performed in clinical populations, even though it has been recognized that declining cognition and psychiatric disease may not be linear and may depend on multiple interacting factors, including structural and functional connectivity (Jones et al., 2016; Schoonheim, Meijer, & Geurts, 2015; Stam, 2014; van Os, Guloksuz, Vijn, Hafkenscheid, & Delespaul, 2019). Study designs are needed that allow assessment of where any patient with a neurological or psychiatric disease is on a hypothesized group-level trajectory of brain network change.

As a feasible way forward, we propose to complement studies using single time points and large samples in future case-control studies of network topology with longitudinal studies that are tailored to the patient population at hand. Such studies should start off with a double baseline at two consecutive moments, with the interval determined using information about the disease at hand: Highly progressive diseases, such as high-grade glioma, should be sampled at shorter intervals than more stable syndromes, such as mild cognitive impairment as a prodromal phase of Alzheimer’s disease. In order to establish multimodal network trajectories, we do not only consider network topology at the absolute first time point, but also assess whether multimodal network topology is in the process of altering in a meaningful way between the first and second baseline within each patient. Collecting data at the same interval in matched, healthy controls should allow for adequate correction for variation between measurements due to measurement error, artifacts, modality-specific noise, and so on. The remaining effects may be interpreted as disease-specific dynamics, reflecting possible inflection points of the individual patient on the hypothesized group-level trajectory. This double baseline would then ideally be followed by longitudinal sampling of the entire disease course, allowing for further characterization of multimodal network changes, possibly going hand in hand with functional decline. Ultimately, this type of “trajectory” would allow us to better understand (cognitive) symptoms in disease, but more importantly may offer new avenues for prediction and treatment of decline.

### What is needed to use brain network characteristics as biomarkers?

Damage to the structural and functional connectome plays an important role in a variety of brain disorders. A major goal of disease connectomics is to map commonalities and differences across disorders, but also within patients, to facilitate diagnosis and track disease progression. Expanding study designs from examining single disorders to sets of disorders may reveal general principles of brain vulnerability, and specific and shared multimodal connectome pathology across diseases (Cauda et al., 2018; Crossley et al., 2014). However, there are yet few signs that structural and/or functional brain network analysis may in fact change clinical practice (Castellanos, Di Martino, Craddock, Mehta, & Milham, 2013; Fornito, Zalesky, & Breakspear, 2013). For example, whereas both visual and spectral analysis of EEG and MEG are informative for the diagnosis and prognosis of certain dementia types (Engels et al., 2016; Gouw & Stam, 2016; Gouw et al., 2017; Olde Dubbelink et al., 2014), functional connectivity and network characteristics seem to add relatively limited clinical value to standard clinical brain imaging (Dauwan et al., 2016; Nissen et al., 2018).

The first clinical application of brain network analysis would be the development of a biomarker: “A characteristic that is objectively measured and evaluated as an indicator of normal biological processes, pathogenic processes, or pharmacologic responses to a therapeutic intervention” (Biomarkers Definitions Working Group, 2001, p. 91). Structural and functional brain network characteristics need to be sufficiently reliable, reproducible, sensitive, and specific to serve as biomarkers. An increasing number of studies report the sensitivity of multimodal brain network characteristics to differentiate between patients and controls (Ding et al., 2013; Vecchio et al., 2018). An important and easily implemented step towards adequate investigation of brain network measures as biomarkers is to consistently report reliability, reproducibility, sensitivity, and specificity of any measure in classifying patients and controls, instead of only reporting statistically significant group differences. Somewhat more difficult, but possibly influential in this context, is creating a large database with all (multimodal) data from both healthy subjects and different patient populations in order to crosslink different investigations.

Another direction is to use brain stimulation techniques as controlled network perturbations, in order to understand the dynamic adaptation of brain networks in healthy and disease states and thus contribute to clinical translation. For instance, TMS can be used to either infer reversible “virtual lesions” in healthy controls (Klomjai, Katz, & Lackmy-Vallée, 2015) or ameliorate brain dysfunction, such as treatment of major depression disorder (Perera et al., 2016). Recent work has determined clinically useful localized targets, based on predominantly functional network topology, that elicit consistent, predictable behavioral responses and treatment effects (Fox et al., 2014; Fox, Liu, & Pascual-Leone, 2013; Weigand et al., 2018). Moreover, it is becoming clear that the functional network topological profile of the target region is predictive of individual patients’ clinical outcome, for instance in patients with Parkinson’s disease (Koirala et al., 2018) or depression (Downar et al., 2014). The success of translation of network neuroscientific approaches to clinical practice may in this context be facilitated by the controlled perturbation achieved by neurostimulation, which is more easily understood and tested than for instance disease progression or systemic treatments.

### Can we use network neuroscience for disease classification?

Network neuroscience may help to classify brain diseases. Current neurological and especially psychiatric disease classifications are often based on symptom co-occurrence, much more than strict knowledge of pathophysiology. Previously separate disease entities may actually share important pathology, like alpha-synuclein deposition across neurodegenerative diseases (Zhang, Nie, & Chen, 2018). Instead of investigating the structural and functional network correlates of the current classification of disease, we argue that comparison of multimodal network patterns within and across classes may bring new insight into (shared) clinical trajectories and potential intervention strategies.

Sets of structural and/or functional network characteristics may be combined to classify patient groups using machine-learning algorithms, such as random forest algorithms (van Diessen, Otte, Braun, Stam, & Jansen, 2013). These algorithms have the advantage that they provide indications of how much each network feature contributes to the classification result, allowing better insight into specific versus general brain disease mechanisms. For example, approximately 50% of variance in different dimensions of psychopathological symptoms that are notoriously difficult to quantify, such as psychosis, mood, and fear, may be explained by only small sets of functional brain network characteristics (Xia et al., 2018).

Integration of network neuroscience in projects such as the Research Domain Criteria (RDoC) may bring further insight into the specificity of structural and functional network alterations in relation to symptom and behavioral domains, treatment response, and prognosis (Insel et al., 2010). The lack of clinical yield when it comes to network neuroscience may lie in the mismatch between rigid diagnostic constructs based on groups of signs and symptoms, and a de facto overlap (or continuum) across diagnostic classes, which share etiological factors, polygenic risks, and comorbidities (Crocq, 2018). Moreover, diagnoses such as schizophrenia or bipolar disorders group patients together that are highly heterogeneous in terms of clinical manifestation, treatment response, and, possibly, pathophysiological mechanisms. The RDoC represents a paradigm shift in this sense, proposing to map individuals in terms of functional domains (e.g., cognitive systems) and related constructs (e.g., working memory) on a full range of variations independent from classic diagnostic boundaries, and spanning the normal (healthy) to abnormal (pathological) spectrum (Cuthbert & Insel, 2013; Insel et al., 2010). Network neuroscience can bring a fundamental contribution to the RDoC paradigm, by defining robust measurements (such as connectivity and topological measures) across levels of analysis, and by integrating those levels of analysis into a unified object of investigation, for example with multilayer network models (Braun et al., 2018). On the one hand, this line of research can identify dimensional and neurobiological constructs that cut *across* diagnostic classes. For example, patterns of functional segregation in the brain network identify psychopathological dimensions across psychiatric groups (Xia et al., 2018). On the other hand, the synergy between RDoC and network neuroscience may help in identifying valid phenotypes *within* diagnostic classes. For example, measures of functional connectivity within brain subnetworks identify subtypes of depression (Drysdale et al., 2017), which could define optimal targets for treatment selection (Furman & Trivedi, 2019) and personalized intervention and prognosis (Cocchi & Zalesky, 2018). Clustering algorithms for (multilayer) patient networks, where edges represent multidimensional similarity between individuals, could also deliver meaningful subtypes within the RDoC framework (Pai & Bader, 2018; Stefanik et al., 2018).

Finally, psychiatric symptoms may be understood from multiple levels of explanation, based on, for example, cultural, social, brain imaging, and molecular information (Hugdahl & Sommer, 2018). Multilayer network approaches may help to come to a conceptual integration of these levels of information (Gosak et al., 2018). This framework may also include novel approaches such as network analysis of symptoms as well as digital tracing of interaction patterns, termed “digital phenotyping” (Lydon-Staley, Barnett, Satterthwaite, & Bassett, 2018), which quantifies interactions with digital devices, providing novel signatures of psychopathology.

### Can we systematically bridge the gap between brain network interventions in silico and in vivo?

Neurological and psychiatric diseases are dynamic in various dimensions, as their symptomatology and multimodal brain network topology change over time. Any intervention in these diseases is therefore aimed at somehow halting or reversing the pathological multimodal network change that has occurred or is imminent. There are many ways to modify brain structure and function (e.g., surgery, medication, [non]invasive stimulation, cognitive therapy), but at present, interventions are usually performed without considering brain connectivity, network embedding, or plasticity (see Section I). This has various potential disadvantages: (a) Interventions may not be as efficient as they could be (Horn et al., 2017; Sale, Mattingley, Zalesky, & Cocchi, 2015); (b) Unexpected adverse effects may occur because of the complex network (re)organization of the human brain, for instance because of time-dependent effects (Cocchi et al., 2016); (c) Clinical trial and error to find optimal treatment is slow, burdensome, and expensive; and (d) The reasons why a certain intervention does or does not work remain unclear (Sale et al., 2015).

Computational network neuroscience has been used to model the impact of different types of pathology and disease stages on the structure and functioning of brain networks (Proix, Bartolomei, Guye, & Jirsa, 2017). An appealing next step is simulation of treatment effects before putting them in practice (Hughes’ “demonstration”): a kind of “virtual trial” (de Haan et al., 2017). An example of this approach has been reported for Alzheimer’s disease: In a computational model that coupled 78 neural mass models according to human, DTI-based topology, damage was applied to the network based on the local levels of neuronal activity (“activity-dependent degeneration”). Simultaneously, virtual interventions that applied different levels of enhancement or suppression of neuronal excitability were compared for their ability to maintain normal network topology over time (de Haan et al., 2017). This led to specific predictions for the optimal stimulation protocol, which can be verified in vivo.

**Figure 2.**
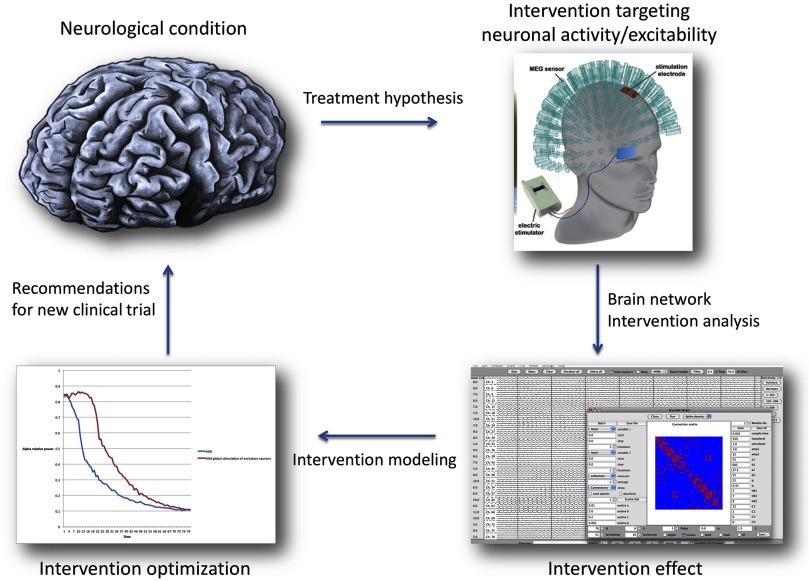
Using in silico and in vivo experiments to advance interventions. A tentative flowchart for the integration of network intervention modeling and clinical studies. Systematic intervention modeling produces predictions for clinical experiments, for example, by altering neuronal excitability and testing large-scale network consequences. Vice versa, observed treatment effects can be used to validate and improve model predictions. This mutually reinforcing approach can improve and speed up treatment development, keeping patient burden at a minimum while providing more insight into treatment success or failure.

In order to cross the barrier towards use in clinical practice, we need to find a method to match theoretical and practical treatment strategies. In nonhuman experiments, combinations of neuronal stimulation and accurate network consequence acquisition and analysis produce fascinating new insights into the organizational principles of the brain (Gollo, Roberts, & Cocchi, 2017; Wang, Hutchings, & Kaiser, 2015). Feasible analogues in humans could be transcranial direct current stimulation (tDCS), transcranial alternating current stimulation (tACS), transcranial focused ultrasound (tFUS), or the previously described TMS: stimulation techniques that alter neuronal excitability, and thereby influence functional network properties (Sale et al., 2015). Since tDCS can be performed during MEG recording, we have a way of simultaneously altering and recording large-scale functional networks. The challenge is then to find reliable ways to produce (and reproduce) desired network manipulations, and turn successful virtual interventions into real ones (see Figure 2).

### Are evolution and dissolution driving factors in disease connectomics?

An evolutionary approach with network neuroscience may aid in understanding the biology of disease: Comparing the topological organization of the human structural and functional brain network to that of other species may enhance our understanding of evolutionary processes that have shaped human brain wiring (Buckner & Krienen, 2013). Comparative connectomics may further our understanding of human brain network topology and its implications for functioning in both health and disease (Rilling & van den Heuvel, 2018; van den Heuvel, Bullmore, & Sporns, 2016). A hypothesis that has been posited for human-specific diseases is that “the evolution of brain wiring in support of complex brain function in humans may have increased the vulnerability to brain dysfunction in disease” (van den Heuvel et al., 2018, p. 2). Schizophrenia for example has been suggested to relate to increased evolutionary pressure on structural brain connectivity in the human brain (Burns, 2004; van den Heuvel et al., 2018), arguably increasing the vulnerability of the human brain to various brain disorders (Gollo et al., 2018).

**Figure 3.**
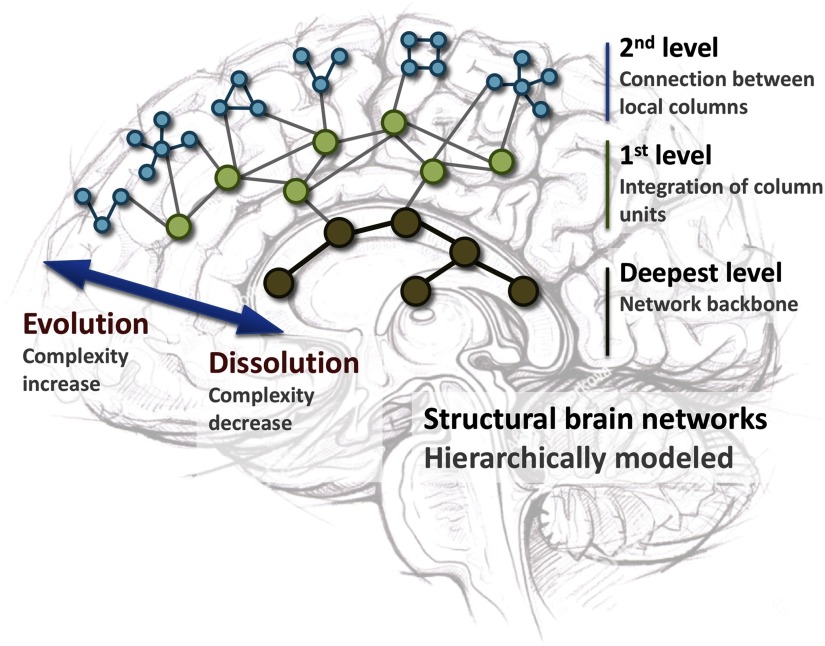
Schematic representation of evolution versus dissolution as a theoretical framework towards understanding epilepsy. Structural brain networks are hierarchically ordered from 100 million cortical minicolumns and 1–2 million cortical columns to distributed column groups and deeper underlying subcortical network structures. Because of the number and interconnectivity of columns, complexity is highest at the cortical column connectivity level and decreases at lower topological levels, namely at the levels of integration of columns and the deeper network backbone. Complexity is defined here as the relative independence of subnetworks and their properties from the integrative network properties at large. An important hallmark of many network disorders is a shift in the network complexity, which is characterized by evolutionary processes of rewiring that increase complexity and network dissolution that decreases complexity.

Using a pathological developmental view, loss of organization of the brain network may be investigated using mapping of dissolution as opposed to evolution. This framework maps end-stage network configurations, irrespective of timescales, in terms of evolution versus dissolution. An obvious example of a relevant disease population to do so is dementia, in which different pathological entities lead to a final common pathway of network dissolution. Another example is epilepsy, wherein the concept of a singular focus has shifted to a more widespread interconnected epileptogenic network (Englot, Konrad, & Morgan, 2016; Kramer & Cash, 2012; van Diessen, Diederen, Braun, Jansen & Stam, 2013). Seizures may recur several years after initially successful epilepsy surgery, possibly because of aberrant plasticity and newly grown connections due to ongoing epileptic activity from (microscopic) parts of the network. John Hughlings Jackson (1835–1911), the founder of contemporary epileptology, already viewed epilepsy as a network disease (Jackson, 1884). He saw the brain as a hierarchically ordered construction of connected brain centers, in which the complexity of connections, due to an exponential growth in network nodes and additional layering, increases when we move from deeper regions toward the neocortex. Hughlings Jackson then labeled this increase in complexity as evolution and the decrease as dissolution (see Figure 3). According to his theory, brain disorders originated not from malfunctioning of one or two specific brain centers, but mainly from deterioration of connections between multiple centers. Epilepsy was then characterized by dissolution of brain networks, so a reversed evolution with a reduction in connection complexity. With this theory he introduced an alternative framework that differed from the, at that time, dominant, binary all-or-nothing concept and preoccupation with regional pathology. Although it has been abandoned for decades, network analysis of longitudinal neuroimaging and physiological datasets may be able to assess the value of understanding evolution and dissolution of networks in the clinical setting of epilepsy.

Statistical models that allow the mapping of evolution-dissolution in (disease) networks are the exponential random graph model (Sinke, Dijkhuizen, Caimo, Stam, & Otte, 2016) or topological motifs morphospace mapping (Morgan, Achard, Termenon, Bullmore, & Vértes, 2018). Both techniques decompose the network in easy to understand building blocks. In addition, the morphospace maps the networks into a common space, allowing the quantification of network relationships over time and the driving forces behind network evolution or dissolution. This approach may also help to assess whether already changed network topologies, for example because of years of suffering from medication-refractory epilepsy, remain vulnerable to disease despite initial treatment.

## CONCLUSION

We have identified a set of questions that may guide future research. Two main themes deserve further attention: (a) the methodological challenges of brain network analysis and (b) the translation of findings to gain understanding of neurological and psychiatric diseases and ultimately improve clinical practice. We have delineated a number of routes forward with respect to these themes, which are without doubt also being explored concurrent to the writing of this review piece.

It is worth mentioning in this context that progress might not occur through experimentation within a settled paradigm or explanatory framework, or according to a tradition of investigation, which Thomas Kuhn termed “normal science” in his seminal work on scientific revolutions (Kuhn, 1962). In (clinical) network neuroscience, such tradition may relate to step-by-step exclusion of hurdles standing in the way of progress, instead of fundamentally challenging the way we investigate brain networks. We therefore invite the field of network neuroscience to not only address obvious hurdles towards full fruition of clinical network neuroscience, but also remain open to unexpected thoughts and ideas. Or, to speak with Paul Feyerabend: *Anything goes*.

## AUTHOR CONTRIBUTIONS

All authors contributed in the conceptualization and writing of the manuscript. Linda Douw and Edwin van Dellen reviewed and edited contributions of the other authors, merged these contributions into an original draft, and revised the manuscript based on peer-review. Linda Douw: Conceptualization; Writing – original draft; Writing – review & editing. Edwin van Dellen: Conceptualization; Writing – original draft; Writing – review & editing. Alida A. Gouw: Conceptualization; Writing – original draft. Alessandra Griffa: Conceptualization; Writing – original draft. Willem de Haan: Conceptualization; Writing – original draft. Martijn van den Heuvel: Conceptualization; Writing – original draft. Arjan Hillebrand: Conceptualization; Writing – original draft. Piet Van Mieghem: Conceptualization; Writing – original draft. Ida A. Nissen: Conceptualization; Writing – original draft. Willem M. Otte: Conceptualization; Writing – original draft. Yael D. Reijmer: Conceptualization; Writing – original draft. Menno M. Schoonheim: Conceptualization; Writing – original draft. Mario Senden: Conceptualization; Writing – original draft. Elisabeth C.W. van Straaten: Conceptualization; Writing – original draft. Betty M. Tijms: Conceptualization; Writing – original draft. Prejaas Tewarie: Conceptualization; Writing – original draft. Cornelis J. Stam: Conceptualization; Writing – original draft.

## FUNDING INFORMATION

Linda Douw, Netherlands Organization for Scientific Research (NWO) Vidi grant, Award ID: 198.015. Edwin van Dellen, The Netherlands Organization for Health Research and Development (ZonMW) GGZ fellowship, Award ID: 60-63600-98-711. Edwin van Dellen, UMC Utrecht Clinical Research Talent Fellowship. Award ID: NA Alessandra Griffa, Swiss National Science Foundation, Award ID: SNSF #P2ELP3_172087. Martijn van den Heuvel, ALW open, Award ID: ALWOP.179; Martijn van den Heuvel, NWO Vidi grant, Award ID: 452-16-015. Martijn van den Heuvel, MQ Fellowship. Award ID: NA Ida A. Nissen, Netherlands Epilepsy Fund, Award ID: 95105006. Willem M. Otte, NWO VENI grant, Award ID: 016.168.038. Menno M. Schoonheim, Dutch MS Research Foundation, Award ID: 13-820; Mario Senden, EU Horizon 2020 program, Q2 Award ID: 737691-HBP SGA2. Betty M. Tijms, ZonMW, Award ID: 73305056.

## TECHNICAL TERMS

Multistability: Coexistence of multiple stable attractors in the state space of the system or network.
Metastability: *Nonlinear dynamics definition*: The dwelling tendency of trajectories in phase space without convergence to an attractor (or remnants of attractors). (This definition is used in the context of this manuscript.) *Network science definition*: Quasi-stable state of the epidemics in an SIS model beyond the epidemic threshold. (This definition is mentioned here only to avoid confusion;it is not used in the context of the current manuscript.)
Attractor: Subspace in phase space to which a trajectory converges for certain initial conditions.
Limit cycle: An isolated periodic oscillator, which is a solution for a dynamical system that repeats itself in time.
Chaos: A periodic long-term behavior in a deterministic system that exhibits sensitive dependence on initial conditions.
Fixed point: A trajectory of a dynamical system that does not change in time. It is also often called an equilibrium or steadystate.
